# Dioscin augments HSV-tk-mediated suicide gene therapy for melanoma by promoting connexin-based intercellular communication

**DOI:** 10.18632/oncotarget.13655

**Published:** 2016-11-26

**Authors:** Jianyong Xiao, Guangxian Zhang, Bin Li, Yingya Wu, Xijuan Liu, Yuhui Tan, Biaoyan Du

**Affiliations:** ^1^ Department of Biochemistry, Guangzhou University of Chinese Medicine, Guangzhou 510006, China; ^2^ Department of Pathology, Guangzhou University of Chinese Medicine, Guangzhou 510006, China

**Keywords:** dioscin, melanoma, bystander effect, suicide gene, connexin

## Abstract

Suicide gene therapy is a promising strategy against melanoma. However, the low efficiency of the gene transfer technique can limit its application. Our preliminary data showed that dioscin, a glucoside saponin, could upregulate the expression of connexins Cx26 and Cx43, major components of gap junctions, in melanoma cells. We hypothesized that dioscin may increase the bystander effect of herpes simplex virus thymidine kinase/ganciclovir (HSV-tk/GCV) through increasing the formation of gap junctions. Further analysis showed that dioscin indeed could increase the gap junctional intercellular communication in B16 melanoma cells, resulting in more efficient GCV-induced bystander killing in B16^tk^ cells. By contrast, overexpression of dominant negative Cx43 impaired the cell-cell communication of B16 cells and subsequently weakened the bystander effect of HSV-tk/GCV gene therapy. *In vivo*, combination treatment with dioscin and GCV of tumor-bearing mice with 30% positive B16^tk^ cells and 70% wild-type B16 cells caused a significant reduction in tumor volume and weight compared to treatment with GCV or dioscin alone. Taken together, these results demonstrated that dioscin could augment the bystander effect of the HSV-tk/GCV system through increasing connexin-mediated gap junction coupling.

## INTRODUCTION

Melanoma is a malignant skin cancer, which has drawn much concern for its high mortality rate and increased incidence in recent decades [1]. Due to the chemoresistance and rapid metastasis of melanoma tumors, conventional treatments by surgical removal, chemotherapy and radiotherapy have provided unsatisfactory outcomes for patients. Therefore, targeted gene therapy currently is recommended for patients with melanoma. Clinical trials have demonstrated the feasibility and safety of gene therapy against malignant melanoma [2].

The goal of gene therapy targeted to melanoma cells is to transfer tumor suppressor genes or to inactivate aberrant oncogene expression. However, the major limitation of gene therapy is the efficiency of gene transfer. Interestingly, studies of suicide gene transfer strategies showed that despite low gene transfer efficiency into tumor cells, the magnitude of the cell killing far exceeded what would be expected based on transfection efficiency. The so-called bystander effect renders suicide gene therapy as a highly promising strategy. The most widely used suicide gene in clinical trials is the HSV-tk gene. After this gene is introduced into tumor cells, patients are given the drug GCV, which is an acyclic nucleoside analogue. When phosphorylated by HSV-tk, GCV incorporates into DNA, resulting in the termination of DNA elongation during the S-phase of transduced tumor cells. Using this strategy, a significant anti-tumor effect was initially demonstrated in a B16 melanoma model [3]. As only limited efficacy of this strategy has been observed in humans, however, a better strategy to improve the bystander killing effect is urgently needed.

The bystander effect described above was shown to be mainly caused by the cell-cell transfer of toxic substrates [4], and increasing the intercellular communication can enhance the killing effect in suicide gene therapy [5]. Among the different types of cell-cell interactions, gap junctional intercellular communication **(**GJIC) is the major mechanism by which cells can directly exchange small cytoplasmic hydrophilic metabolites. This transfer occurs via intercellular channels gathered into gap junction plaques, which are comprised of juxtaposed transmembrane hemichannels or connexins provided by adjacent cells. Each connexin is composed of six protein subunits. Cx26 and Cx43 were reported to be key components in the gap junctions of melanoma cells, but they were found to be expressed only at low basal levels, rendering the cells GJIC-deficient.

Dioscin is a naturally occurring glucoside saponin present in roots of wild yam (*Dioscorea villosa*) [6]. It possesses a wide range of health beneficial properties and has been used in traditional medicine for its anti-inflammation, lipid-lowering and hepatoprotection effects [7–9]. Apart from the traditional application of dioscin, increasing lines of evidence that cover a wide range of cancer cell lines suggested that dioscin may exert anti-cancer effects via cell cycle arrest and induction of apoptosis [10, 11]. However, investigations of the anti-cancer effects of dioscin *in vivo* are rare.

Recently, we found that a low concentration of dioscin could upregulate Cx26 and Cx43 in melanoma B16 cells. Following up on those results, we demonstrated here that dioscin could intensify the bystander killing efficiency of the HSV-tk/GCV system in B16 cells by promoting GJIC *in vitro*. The *in vivo* synergistic inhibition of B16 cells by dioscin and the HSV-tk/GCV system was also observed.

## RESULTS

### Dioscin increases GJIC of B16 melanoma cells

**Figure 1:**
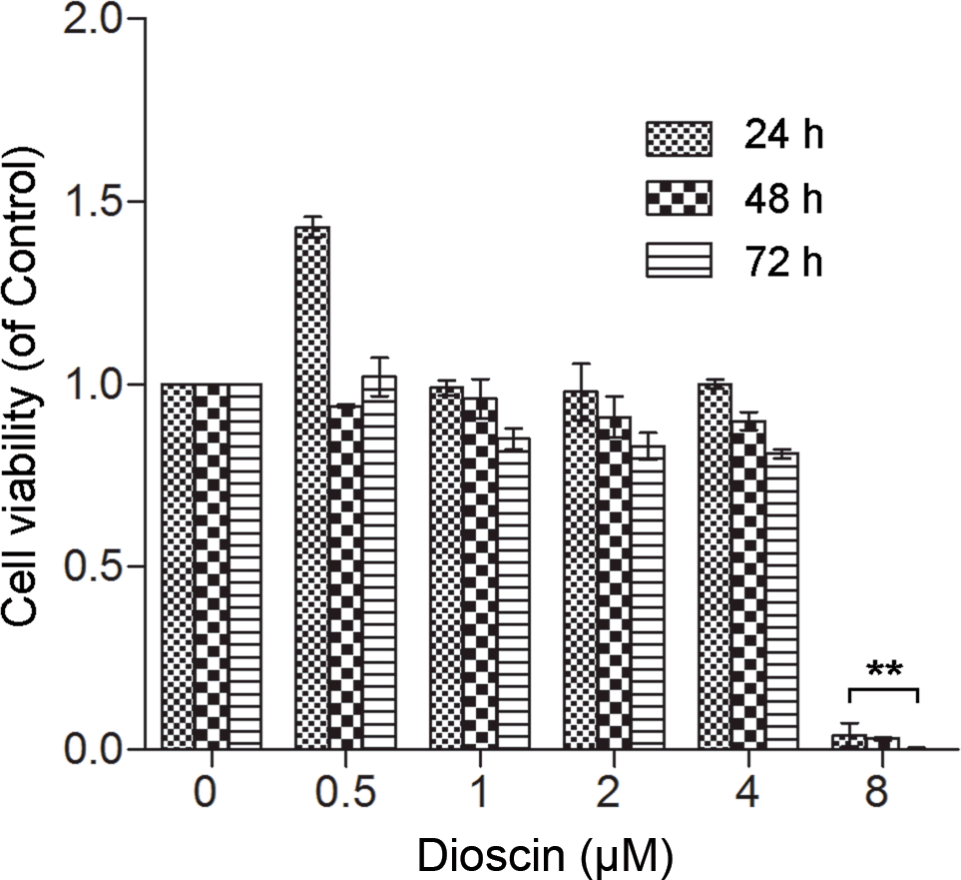
Effect of dioscin on B16 cell viability B16 cells were seeded at a density of 1 × 10^4^ cells in 96-well culture plates and treated with dioscin (0, 0.5, 1, 2, 4 and 8 μM) for 24, 48 and 72 h. Cell viability was examined by the MTT assay. **P < 0.01, compared with control.

To test the effect of dioscin on GJIC of B16 cells, we first performed the MTT assay to determine the applicable concentration of dioscin. As seen in Figure 1, low concentrations of dioscin (≤ 4 μM) had no significant effect on B16 cell viability, whereas 8 μM dioscin resulted in a high level of cytotoxicity in B16 cells.

**Figure 2:**
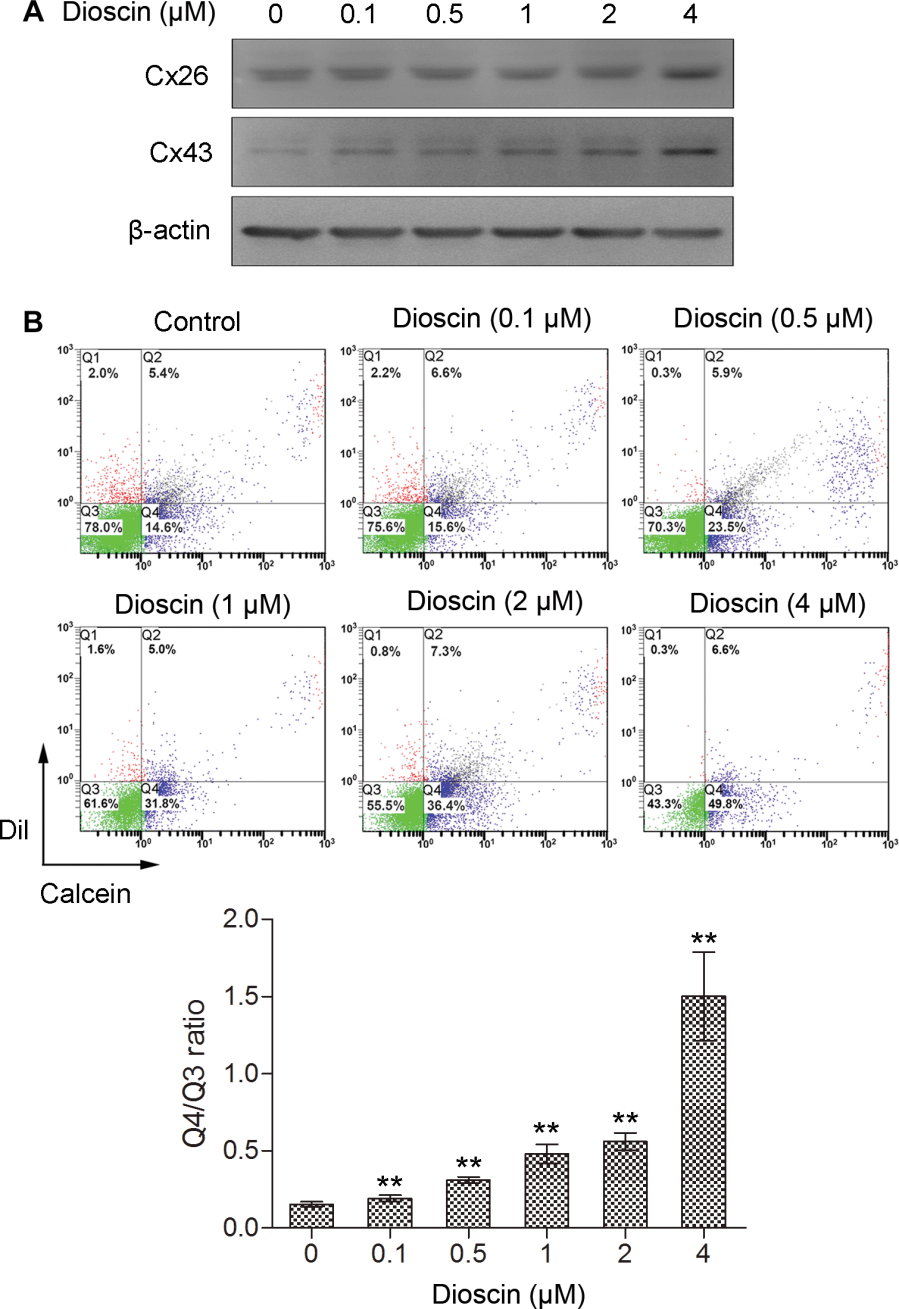
Increase of GJIC by dioscin in B16 melanoma cells (**A**) Upregulation of Cx26 and Cx43 proteins in dioscin-treated B16 cells examined by immunoblotting (**B**) Promotion of GJIC by dioscin in B16 cells, as measured by fluorescent dye transfer assay. Q2: DiI and Calcein double-positive cell populations (donor cells); Q4: Calcein-positive cells (recipient cells). The ratio of the B16 cell number in Q4 to that in Q3 (double negative cells) was used to evaluate GJIC function. The lower panel shows the quantification from three independent experiments. **P < 0.01, compared with control.

Next, we treated B16 cells with low concentrations of dioscin (0.1, 0.5, 1, 2 and 4 μM) and examined the expression levels of Cx26 and Cx43, which are the most predominant gap junction proteins in melanoma cell lines. Western blot analysis indicated that the expression of Cx43 was upregulated in a dose-dependent manner after dioscin treatment. Cx26 was also highly expressed in B16 cells under dioscin treatment (4 μM), indicating that exposure of these cells to dioscin could upregulate the expression of connexins (Figure 2A).

To determine whether dioscin could increase the formation of gap junctions in B16 cells, a fluorescent dye transfer experiment was conducted to assess GJIC following treatment with this drug. As shown in Figure 2B, Q2 indicates the donor cells (pre-labeled with DiI and Calcein AM); meanwhile, Q4 indicates the recipient cells that received Calcein from donor cells through gap junctions, and Q3 denotes the DiI and Calcein AM double-negative cells. Therefore, the ratio of B16 cell numbers in quadrant Q4 (Calcein-positive) to that of Q3 (fluorescence dye-negative cells) was used to evaluate the transfer of Calcein as an indication of GJIC function. The Q4/Q3 ratio was 0.15 in the control group. In comparison, after exposure of B16 cells to different concentrations of dioscin (0.1, 0.5, 1, 2 and 4 μM), the ratios of Q4 to Q3 were 0.19, 0.31, 0.48, 0.56 and 1.50, respectively. The Q4/Q3 ratios of experimental groups were higher than that of the control (**P < 0.01), indicating that cell-to-cell spread of Calcein was more efficient after dioscin treatment. The fluorescence dye transfer analysis demonstrated that dioscin could dose-dependently enhance GJIC among the B16 cells.

### Dioscin enhances the bystander effect of HSV-tk/GCV-mediated gene therapy in B16 cells

**Figure 3:**
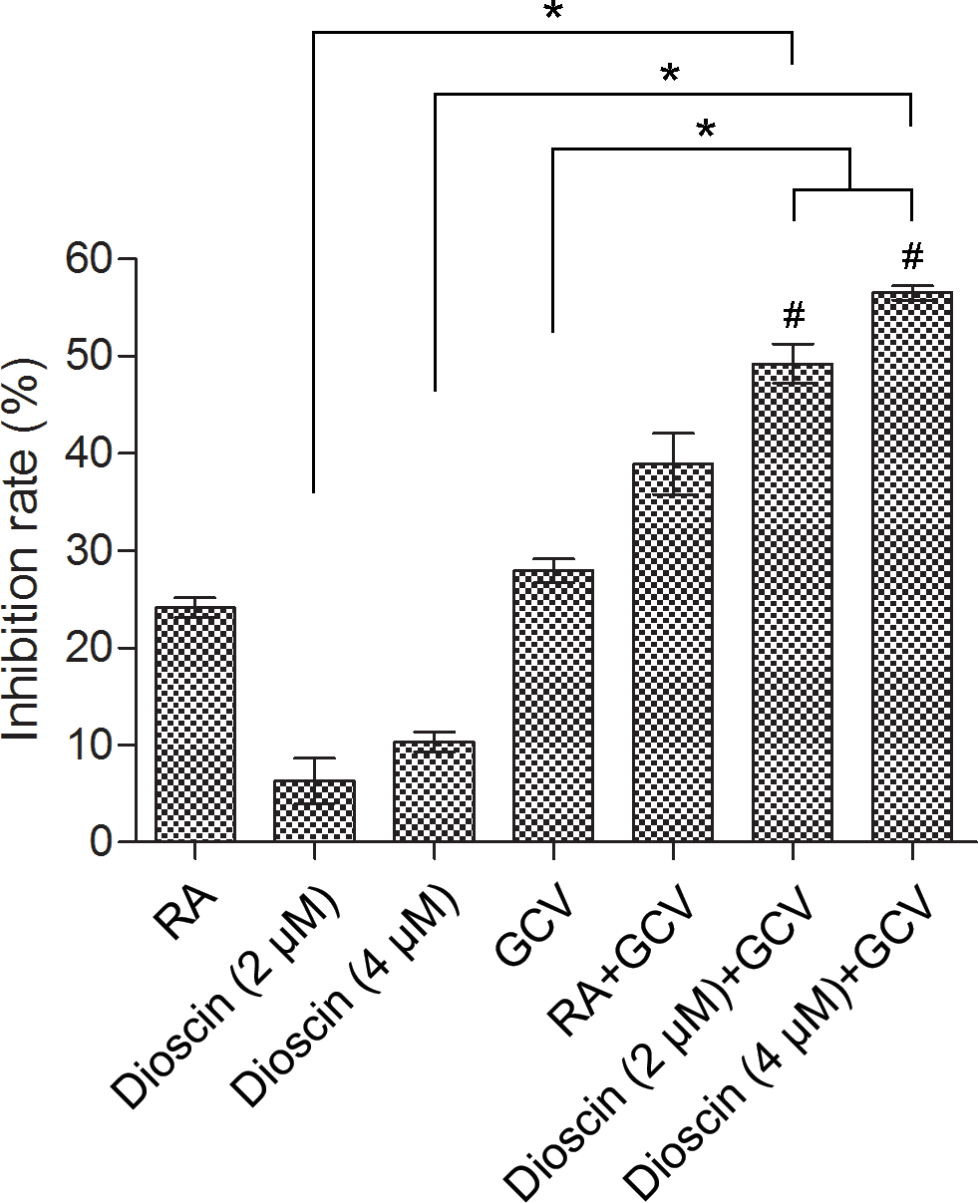
Enhanced growth inhibition of mixture of B16tk-GFP cells and B16RFP cells by dioscin plus GCV combination Stable B16^tk-GFP^ cells were mixed with B16^RFP^ cells at a ratio of 3:7. The mixed cells were treated with 10 μM RA (positive control), dioscin (2 and 4 μM) or DMSO (negative control) for 24 h and then supplemented with or without GCV (15 μM) for an additional 48 h before examination by the MTT assay. *P < 0.05. GCV combined with dioscin (2 and 4 μM) showed a synergistic inhibitory effect on the growth of mixed B16 cells (^#^Q = 1.52 and 1.60, respectively, > 1.15).

The bystander effect of suicide gene therapy is mainly mediated by GJIC. Therefore, we addressed whether dioscin could enhance the HSV-tk/GCV-mediated bystander effect in B16 cells. A co-culture assay was performed in which B16^tk-GFP^ cells and B16^RFP^ cells were mixed at a ratio of 3:7. The mixed cells were co-cultured for 24 h and then treated with 10 μM retinoic acid (RA) as a positive control, GCV (15 μM) or dioscin (2 and 4 μM) alone or the combination of dioscin and GCV for 48 h. Results of the MTT assay indicated that GCV combined with dioscin (2 and 4 μM) caused greater inhibition of mixed B16 cells (49.2% and 56.5%, respectively) compared with GCV (27.9%) or dioscin (2 and 4 μM) (6.3% and 10.3%, respectively) treatment alone (*P* < 0.05; Figure 3). Effects of GCV together with dioscin (2 and 4 μM) were also assessed by calculating the Q values (1.52 and 1.60, respectively), which indicated that this drug combination exerted a synergistic inhibitory effect on the growth of mixed B16 cells (Q > 1.15).

**Figure 4:**
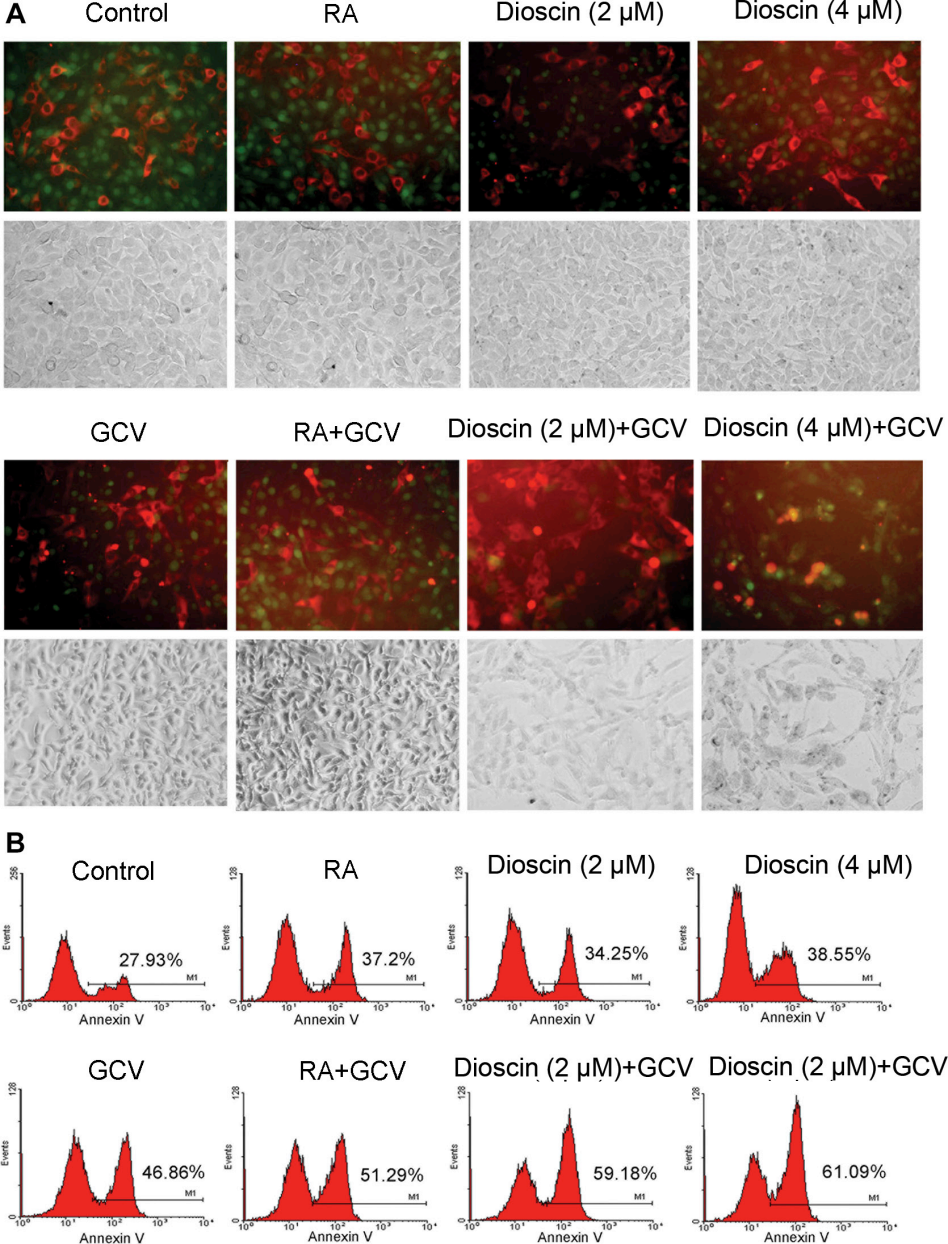
Enhancement of bystander effect of HSV-tk/GCV-mediated gene therapy against B16 cells by dioscin treatment Stable B16^tk-GFP^ cells were mixed with B16^RFP^ cells at a ratio of 3:7 and treated with 10 μM RA (positive control), dioscin (2 and 4 μM) or DMSO (negative control) for 24 h and then supplemented with or without GCV (15 μM) for an additional 48 h. The treated cells were then observed by fluorescence microscopy and also assessed by flow cytometry with annexin V staining. (**A**) Representative fluorescence microscopy images. The aggregation of red fluorescence indicated apoptosis of stable RFP-expressing cells. (**B**) Apoptotic rates of RFP cells were analyzed by flow cytometry.

In parallel, the mixed cells with the same drug treatment as those analyzed in the MTT assay were observed by fluorescence microscopy. As shown in Figure 4A, the aggregation of red fluorescence indicates apoptosis, as RFP is normally expressed in the cytoplasm of living cells. Only a small proportion of B16^RFP^ cells underwent apoptosis when the mixed cells were treated with RA, GCV or dioscin alone. In comparison, the combination treatment of dioscin and GCV resulted in the remarkably increased proportion of apoptotic cells. To quantify these results, the mixed cells were stained with annexin V (green) and then analyzed for apoptosis by flow cytometry. As seen in Figure 4B, GCV treatment alone caused 46.86% of the mixed cells to undergo apoptosis. In contrast, the apoptotic rate was remarkably increased when the mixed cells were cultured with both dioscin (2 and 4 μM) and GCV (59.18% and 61.09%, respectively). The increased apoptosis of HSV-tk-negative RFP cells demonstrated that dioscin enhanced the bystander killing effect of the HSV-tk/GSV-mediated gene therapy in B16 cells.

### Dominant negative mutants of Cx43 attenuate dioscin-induced bystander effect of suicide gene therapy in B16 cells

**Figure 5:**
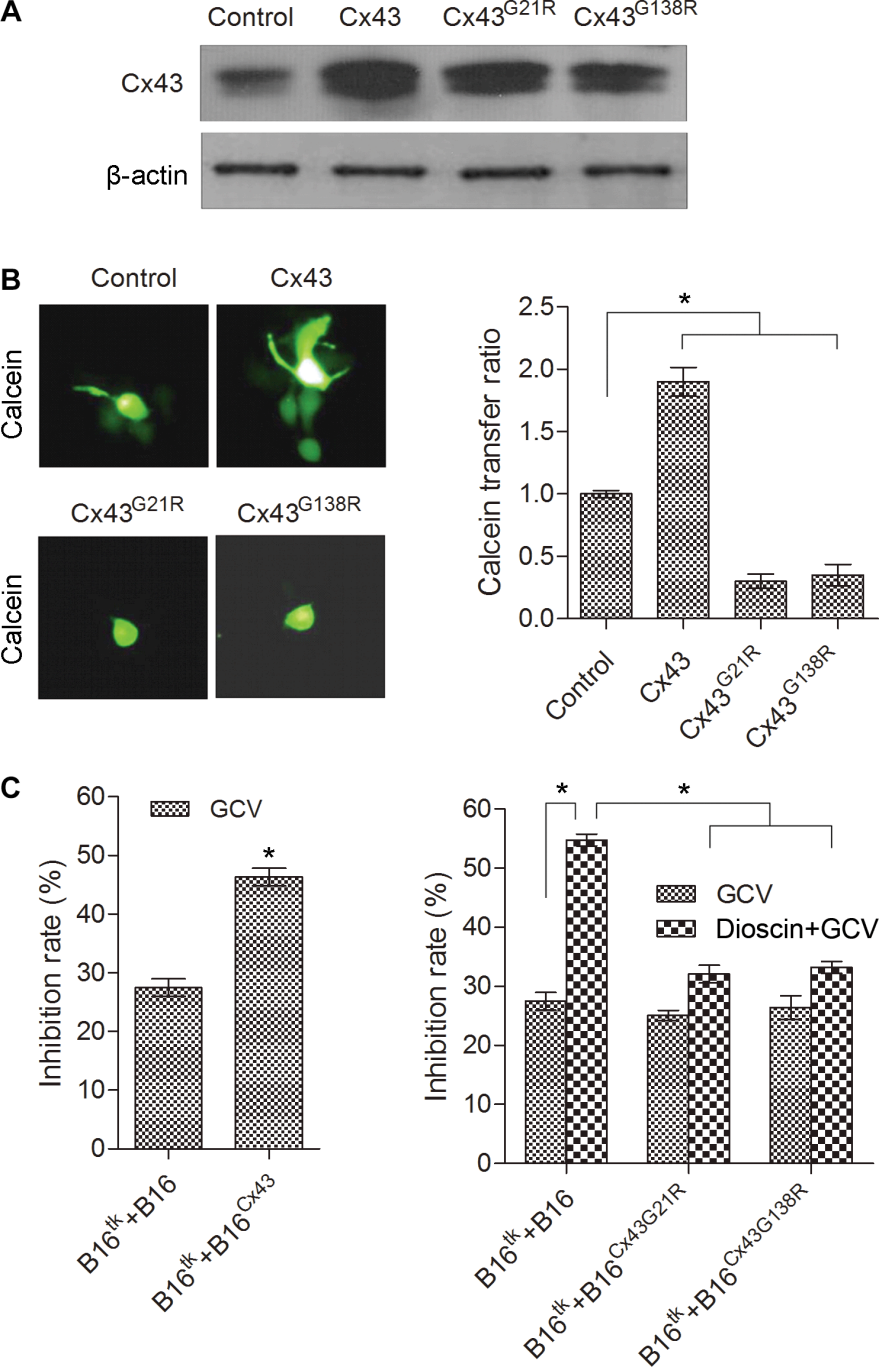
Attenuation of dioscin-induced bystander effect of suicide gene therapy in B16 cells by dominant negative Cx43 (**A**) Expression levels of wild-type Cx43 and mutant Cx43 (Cx43^G21R^ and Cx43^G138R^) correspondingly in B16^Cx43^, B16^Cx43G21R^ and B16^Cx43G138R^ stable cell lines were examined by immunoblotting using an antibody against Cx43. (**B**) Dominant negative mutants of Cx43 (both Cx43^G21R^ and Cx43^G138R^), contrary to over-expression of wild-type Cx43, impaired the GJIC. A 1:100 mixture of donor stable B16 cells (pre-labeled with Calcein AM) and recipient B16 stable cells was co-cultured for 6 h and observed by fluorescence microscopy (left panel). The ratio of green recipient cells (Calcein-positive) vs. donor cells was used to evaluate the transfer of Calcein as an indication of GJIC function. The right panel shows the quantification from three independent experiments. *P < 0.05, compared with control. (**C**) Impact of the gain or loss of Cx43 on the bystander effect of the HSV-tk/GCV gene therapy system against B16 cells. B16^tk^ cells were mixed with HSV-tk-negative B16 cells (B16 cells, B16^Cx43^ cells, B16^Cx43G21R^ cells and B16^Cx43G138R^ cells) at a ratio of 3:7. The mixed cells were treated with GCV (15 μM) alone or the combination of GCV (15 μM) and dioscin (4 μM) for 48 h followed by MTT assay. *P < 0.05.

The expression level of Cx43 was shown above to be significantly upregulated after dioscin treatment. To address whether the increased Cx43 contributed to the dioscin-induced bystander effect of the suicide gene in B16 cells, we constructed three stable B16 cell lines over-expressing wild-type Cx43 (B16^Cx43^), dominant negative mutant Cx43^G21R^ (B16^Cx43G21R^) and dominant negative mutant Cx43^G138R^ (B16^Cx43G138R^), which were confirmed by Western blot (Figure 5A). Next, the function of gap junctions in the different stable B16 cell lines was analyzed by a fluorescence transfer assay, in which donor stable B16 cells pre-labeled with green fluorescence dye-Calcein AM were mixed with unlabeled recipient B16 stable cells at a ratio of 1:100. The mixed cells were co-cultured for 6 h and observed by fluorescence microscopy. The ratio of green recipient cells (Calcein-positive) vs. donor cells was used to evaluate the transfer of Calcein as an indication of GJIC function. Compared with normal B16 cells, B16^Cx43^ stable cells showed a higher fluorescence transfer ratio, as opposed to either B16^Cx43G21R^ or B16^Cx43G138R^ stable cells in which the intercellular fluorescence transfer ratio was dramatically reduced (Figure 5B). The results demonstrated that dominant negative mutants of Cx43 (both Cx43^G21R^ and Cx43^G138R^), contrary to overexpression of wild-type Cx43, impaired the GJIC.

To investigate the impact of the gain or loss of Cx43 on the bystander effect of the HSV-tk/GCV gene therapy system in B16 cells, B16^tk^ stable cells were mixed with HSV-tk negative B16 cells (B16 cells, B16^Cx43^ cells, B16^Cx43G21R^ cells or B16^Cx43G138R^ cells) at a ratio of 3:7. The growth of the mixed cells was examined first in the absence of drug treatment. Except for the suppressed growth rate of the mixture of B16^tk^ cells and B16^Cx43^ cells when co-cultured for 72 h, results of the MTT assay showed no significant difference in the viability between the mixed cells and control B16 cells following 48 or 72 h of culture (data not shown). After exposure to GCV (15 mM), a greater inhibition rate (46.32%) was observed in the mixture of B16^tk^ cells and B16^Cx43^ cells than the control B16 cells (27.46%), B16^tk^ cells mixed with B16^Cx43G21R^ cells (25.07%) or B16^tk^ cells mixed with B16^Cx43G138R^ cells (26.44%). Notably, with the combination treatment with dioscin and GCV, the control group showed more efficient killing (54.78%) compared with the group of B16^tk^ mixed with B16^Cx43G21R^ cells (32.08%) or B16^tk^ cells mixed with B16^Cx43G138R^ cells (33.21%) (Figure 5C). These results demonstrated that Cx43 mediated the bystander killing effect of the HSV-tk/GCV system.

### Dioscin synergistically augments the killing effect of the HSV-tk/GCV system in B16 melanoma cells *in vivo*

**Figure 6:**
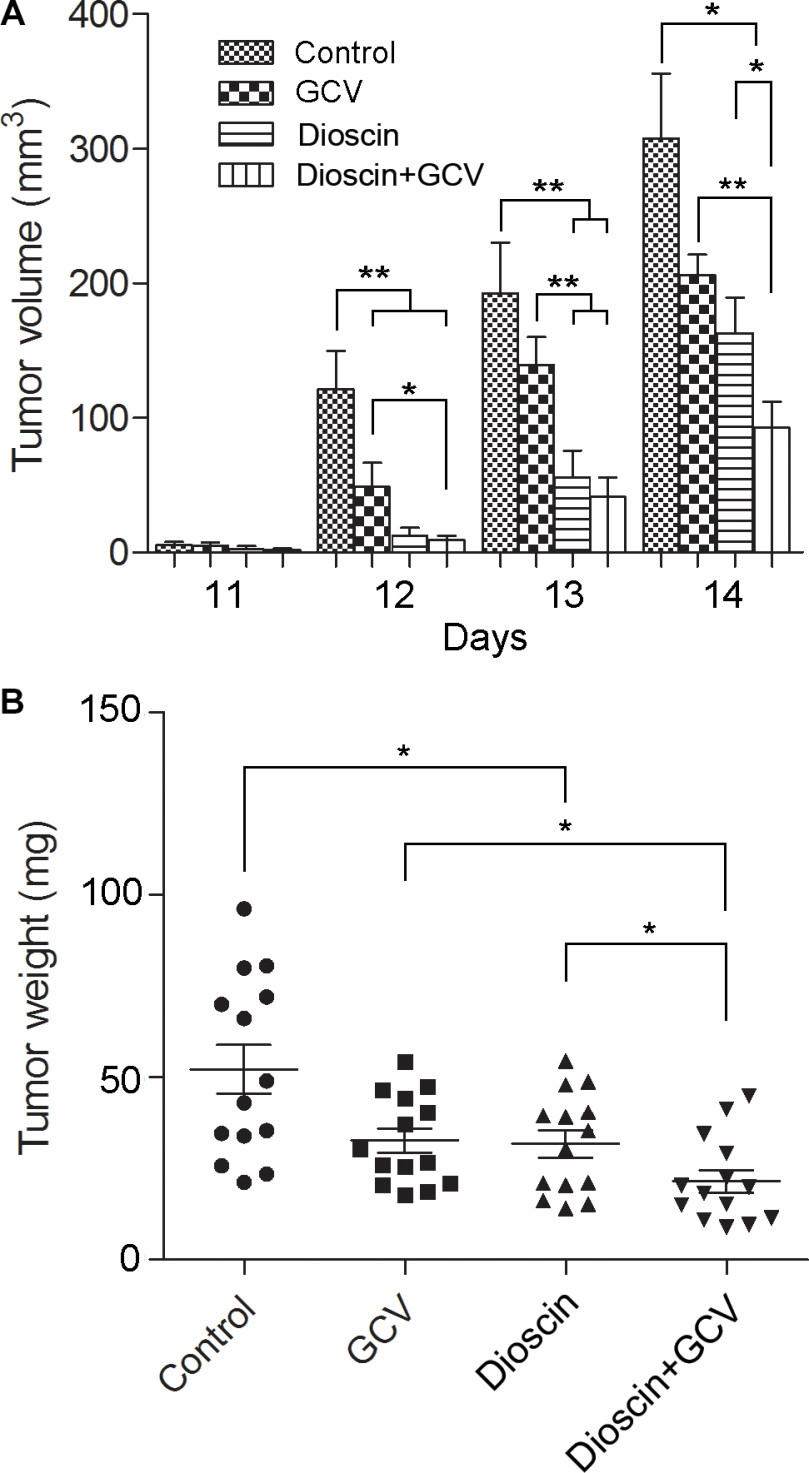
Synergistic inhibition by dioscin and GCV of tumor growth in a mixed population of B16^tk^ cells and wild-type B16 cells *in vivo* Subcutaneous B16 tumors were established in C57BL/6J mice using B16^tk^ cells and wild-type B16 cells mixed at a ratio of 3:7. Mice were then divided into four treatment groups (*n* = 14 mice per group) : control (saline only), GCV only (100 mg/kg·day), dioscin only (50 mg/kg·day), and GCV plus dioscin. (**A**) Tumor volume was measured from day 11, once a day for 4 days. (**B**) Tumor weight was measured on day 14 following treatment. Data are presented as the mean ± standard error. *P < 0.05; **P < 0.01.

To study the impact of dioscin on the bystander effect *in vivo*, a 3:7 mixture of B16^tk^ cells and B16 cells was injected subcutaneously into the flanks of C57BL/6J mice. Mice were then divided into four groups (*n* = 14 per group). The control group received saline only, while drug treatment groups were administered intraperitoneally GCV (100 mg/kg·day) or dioscin (50 mg/kg·day) alone or both dioscin and GCV. The volume and weight of the tumor were measured 14 days after treatment (Figure 6). After completion of the therapy, the impact of the drugs on mouse growth was first evaluated, which showed that the weight of each mouse was not significantly different compared with the control group.

Dioscin was reported to inhibit cancer cell growth and induce apoptosis *in vitro*, but its anti-tumor activity *in vivo* was rarely seen. Here, we found that compared with the mice treated with saline only, mice treated with dioscin alone showed a 47.03% reduction in tumor volume (163.03 ± 26.52 vs. 307.77 ± 47.79, *P* < 0.05) and a 39.24% decrease in tumor weight (317.57 ± 36.74 vs. 522.64 ± 65.96, *P* < 0.05). Although GCV therapy alone caused a certain level of inhibition on tumor growth in the flanks of mice (33.00% reduction in tumor volume, *P* > 0.05; 37.46% reduction in tumor weight, *P* < 0.05), the combinatorial therapy of dioscin and GCV resulted in a 69.95% reduction in tumor volume (92.50 ± 19.70 vs. 307.77 ± 47.79, *P* < 0.05) and a 58.70% reduction in tumor weight (215.86 ± 31.24 vs. 522.64 ± 65.96, *P* < 0.05) compared with the control. Notably, B16-bearing mice treated with dioscin and GCV showed significant inhibition of tumor growth compared with GCV or dioscin treatment alone (*P* < 0.05). Altogether, these data demonstrated that dioscin could synergize with GCV to inhibit the growth of tumor cells *in vivo*.

## DISCUSSION

Clinical trials have shown the feasibility and safety of gene therapy targeting human melanoma [2]. However, the current gene transduction technology cannot deliver the gene of interest to every tumor cell. Thus, the bystander effect is an interesting phenomenon that was expected to overcome the limitations of gene transfer [12]. Transfected cancer cells are thought to exert the bystander killing effect on adjacent cells primarily through gap junctions, the main pathway for cell-cell communication [13]. However, melanoma cells normally have weakened intercellular communication due to the downregulation of connexins that comprise the gap junctions [14]. In this study, we identified that dioscin could induce the upregulation of Cx26 and Cx43, which are the connexin proteins mainly expressed in melanoma cells. By combining dioscin and the suicide gene HSV-tk/GCV system to treat melanoma B16 cells, we observed encouraging anticancer effects both *in vitro* and *in vivo*.

Dioscin is consumed by populations in parts of Asia, Latin America and Eastern Europe as a food ingredient or part of cosmetic formulations [6]. Safety evaluations of dioscin indicated no subchronic toxicity in female rats and slight subchronic toxicity in male rats [15]. Although dioscin was observed to stimulate rat growth-hormone release in a rat pituitary cell culture system [16], here we used the 50 mg/kg concentration of dioscin per day to treat mice for 14 days, which showed no significant effect on the weight of mice (Supplementary Figure S1).

Dioscin has been proposed to be effective against inflammation, obesity and liver fibrosis [7, 8, 17]. Recently, dioscin has gained attention for its anti-cancer effects by induction of cell cycle arrest and apoptosis [10, 11]. In our study, with exposure of B16 cells to a high concentration of dioscin (8 μM), the cell viability was significantly inhibited (Figure 1). *In vivo*, B16-bearing mice treated with dioscin (50 mg/kg·day) showed a dramatic decrease in tumor weight and tumor volume (Figure 6). These data indicated that dioscin exerted anti-cancer effects on B16 melanoma cells.

GJIC participates in the regulation of carcinogenesis. Reduced or aberrant gap junction or connexins expression has been found in many tumors and tumor cell lines [18–20]. The decrease of Cx43 in the early stages of melanoma progression may be important for cells to physically detach from each other [21]. Overexpression of wild-type Cx43 in our study caused the inhibition of the growth of B16 cells after culture for 72 h. The data supported the notion that connexins may act as tumor suppressors [22]. However, Cx43 and Cx26 were also found to be upregulated in melanoma metastatic lesions, which may contribute to the local cell invasion into the surrounding stroma and vasculature [21, 23]. Despite the controversial roles and differential expression of connexins in different stages of cancer progression, overexpression of Cx43 was found to consistently increase the bystander effect of the HSV-tk/GCV suicide gene system through promoting connexin-mediated intercellular communication [24–26]. By contrast, the dominant negative mutants of Cx43 attenuated the intercellular communication of B16 cells and weakened the cellular inhibition rate of dioscin and GCV. These results demonstrated that dioscin and GCV exerted bystander effects through Cx43.

In summary, low concentrations of dioscin were found to increase cell-cell communication of melanoma B16 cells by upregulation of connexins. The combination of dioscin and GCV is expected to be utilized for its potential tumor killing mechanism by synergistically enhancing the bystander effect in the HSV-tk suicide gene system.

## MATERIALS AND METHODS

### Chemicals and reagents

Dioscin (99% pure) was purchased from Ronghe Medical Science and Technology Development Co. (Shanghai, China). GCV (≥ 99% pure) used *in vitro* was obtained from Sigma (Guangzhou, China) and dissolved in PBS before use. For animal experiments, GCV was purchased from Hubei Waterstone Pharmaceutical Co. (Qianjiang, China). Calcein AM and DiI were obtained from Invitrogen (Guangzhou, China).

### Cell lines and cell culture

The murine malignant melanoma cell line B16 was a gift from Zhenhua Xu (HKU, University of Hong Kong, Hong Kong Special Administrative Region of China). By lentiviral infection, the following B16 stable cell lines were established: 1) B16^tk-GFP^ cells (B16 cells stably expressing HSV-tk and enhanced green fluorescent protein); 2) B16^RFP^ cells (B16 cells stably expressing red fluorescent protein); 3) B16^Cx43^ cells (B16 cells stably expressing wild-type Cx43 protein); 4) B16^Cx43G21R^ cells (B16 cells stably expressing Cx43 with mutation at G21R); 5) B16^Cx43G138R^ cells (B16 cells stably expressing Cx43 with G138R mutation). Cells were cultured in RPMI 1640 supplemented with 10% fetal bovine serum (FBS) and 100 U/mL penicillin and streptomycin.

### Double fluorescent dye transfer assay

B16 cells were treated with dioscin for 48 h and divided into two groups, donor cells and recipient cells. The donor cells were digested in a single-cell suspension and pre-loaded with two fluorescent dyes (DiI and Calcein AM) for 30 min. Pre-labeled donor cells were mixed with unlabeled recipient cells at a 1:100 ratio. The level of GJIC was determined by flow cytometry after co-culturing for 6 h. The excitation wavelength was 488 nm, and the emission wavelengths for Calcein and DiI were 525 nm and 575 nm, respectively. DiI (lipophilic red fluorescent dye) does not pass through gap junctions, while Calcein (transferable green dye) passes readily between functionally coupled cells in a gap junction-dependent manner. The extent of dye coupling was defined by the percentage of recipient cells that received Calcein from donor cells. The experiment was repeated three times.

### Western blot for detecting expression of connexin proteins

Ten thousand B16 cells per well were seeded into 6-well plates. Twenty-four hours later, the medium was refreshed and supplemented with dioscin (0, 0.1, 0.5, 1, 2 or 4 μM). After continuous treatment with dioscin for 48 h, drug-treated and untreated cells were washed twice with PBS and lysed in RIPA buffer (0.25 m Tris-HCl pH 6.8, 8% SDS, 1 mM phenylmethylsulfonyl fluoride, 10 mg/mL aprotinin, 1.0 mg/mL leupeptin). The cell extracts were separated by 10% SDS-PAGE and immunoblotted using anti-Cx26 (#T0374; Epitomics), anti-Cx43 (#A0084; Abclonal) or anti-actin (#BM0626; Boster) antibody.

### Influence of dioscin on bystander effect

Stable B16^tk-GFP^ cells were mixed with B16^RFP^ cells at a ratio of 3:7. For the MTT assay, the mixed cells were seeded in 96-well plates (3 × 10^3^ cells per well). On day 2, the culture medium was refreshed and supplemented with 10 μM RA (positive control), dioscin (2 or 4 μM) or DMSO (negative control). Twenty-four hours later, the cells were supplemented with or without GCV (15 μM) for an additional 48 h, followed by analysis using the MTT assay [100 mL (5 g MTT per 1 L serum-free medium)]. The absorbance was measured at 570 nm with an ELISA plate reader, and inhibition was calculated as follows: inhibition rate = 1 - survival rate = (1 - OD_treatment_/OD_control_) × 100%. Each assay was repeated three times (*n*= 4 each time). For analysis of apoptosis, the mixed cells were seeded at a density of 1 × 10^6^ cells in 6-well culture plates and subjected to the same treatment with drugs as above. The treated cells were then observed by fluorescence microscopy. The aggregation of red fluorescent signals indicated apoptosis of stable RFP-expressing cells, mainly caused by the bystander effect of the HSV-tk suicide gene. The apoptosis of treated mixed cells was also assessed by flow cytometry. Briefly, following treatment, the cells were harvested, washed twice with pre-chilled PBS and resuspended in 1× binding buffer at a concentration of 1 × 10^6^ cells/ml. This solution (100 μl) was mixed with 5 μl annexin V-FITC for 15 min, and then 400 μl 1× binding buffer was added. The analysis was carried out using a FACStar cytofluorometer with CXP software.

### Impact of Cx43 on bystander effect

Stable B16^tk^ cells were mixed with wild-type B16 cells, B16^Cx43^ cells, B16^Cx43G21R^ cells or B16^Cx43G138R^ cells at the ratio of 3:7. The mixed cells were seeded at a density of 1 × 10^4^ cells in 96-well culture plates and treated with dioscin (4 μM) for 24 h. Thereafter, the cells were supplemented with or without GCV (15 μM) for an additional 48 h. The viability of the treated mixed cells was examined by the MTT assay. Each assay was repeated three times (*n* = 4 each time).

### Animals

Specific pathogen free C57BL/6J mice weighing approximately 20 g (equivalent numbers of males and females) were purchased from the laboratory animal center at Sun Yat-Sen University and maintained in the animal facility at Guangzhou University of Chinese Medicine. All protocols were approved by the Animal Experimentation Ethics Committee of Guangzhou University of Chinese Medicine in compliance with the recommended NIH guidelines for care and use of animals for scientific purposes.

### *In vivo* experiments

B16^tk^ cells were mixed with wild-type B16 cells at a ratio of 3:7. Two million mixed cells were injected intraperitoneally into the right flanks of C57BL/6J mice. The mice were randomized to four groups (*n* = 14 mice per group), including a saline control group, a group treated with GCV (100 mg/kg·day), a group treated with dioscin (50 mg/kg·day) and a group treated with both dioscin and GCV. Treatment with dioscin was initiated on the day following tumor injection and given for 14 days. Eight days after the tumor injection, GCV (100 mg/kg·day) treatment was started and continued over a 7-day course.

### Statistical analysis

All data were expressed as the mean ± standard error. Data analysis was performed using analysis of variance. When significant differences were found, Fisher's test was used for comparisons among groups. *P* < 0.05 was considered statistically significant.

The Q value was calculated to assess the effect of the combination of drugs as follows: Q = E_AB_/[E_A_ + E_B_ (1-E_A_)], where E_A_, E_B_ and E_AB_ indicate the effects of drug A, drug B and the combination of both drugs, respectively. Q > 1.15 indicates a synergistic effect; Q < 0.85 means an antagonistic effect; and 0.85 < Q < 1.15 denotes an additive effect [27].

## SUPPLEMENTARY MATERIALS



## References

[1] Foletto MC, Haas SE (2014). Cutaneous melanoma: new advances in treatment. An Bras Dermatol.

[2] Sotomayor MG, Yu H, Antonia S, Sotomayor EM, Pardoll DM (2002). Advances in gene therapy for malignant melanoma. Cancer Control.

[3] Vile RG, Hart IR (1993). Use of tissue-specific expression of the herpes simplex virus thymidine kinase gene to inhibit growth of established murine melanomas following direct intratumoral injection of DNA. Cancer Res.

[4] Vile RG, Nelson JA, Castleden S, Chong H, Hart IR (1994). Systemic gene therapy of murine melanoma using tissue specific expression of the HSVtk gene involves an immune component. Cancer Res.

[5] Hattori Y, Maitani Y (2005). Folate-linked nanoparticle-mediated suicide gene therapy in human prostate cancer and nasopharyngeal cancer with herpes simplex virus thymidine kinase. Cancer Gene Ther.

[6] (2004). Final report of the amended safety assessment of Dioscorea Villosa (Wild Yam) root extract. Int J Toxicol.

[7] Wu S, Xu H, Peng J, Wang C, Jin Y, Liu K, Sun H, Qin J (2015). Potent anti-inflammatory effect of dioscin mediated by suppression of TNF-alpha-induced VCAM-1, ICAM-1and EL expression via the NF-kappaB pathway. Biochimie.

[8] Liu M, Xu L, Yin L, Qi Y, Xu Y, Han X, Zhao Y, Sun H, Yao J, Lin Y, Liu K, Peng J (2015). Potent effects of dioscin against obesity in mice. Sci Rep.

[9] Xu T, Zheng L, Xu L, Yin L, Qi Y, Xu Y, Han X, Peng J (2014). Protective effects of dioscin against alcohol-induced liver injury. Arch Toxicol.

[10] Gao LL, Li FR, Jiao P, Yang MF, Zhou XJ, Si YH, Jiang WJ, Zheng TT (2011). Paris chinensis dioscin induces G2/M cell cycle arrest and apoptosis in human gastric cancer SGC-7901 cells. World J Gastroenterol.

[11] Kim EA, Jang JH, Lee YH, Sung EG, Song IH, Kim JY, Kim S, Sohn HY, Lee TJ (2014). Dioscin induces caspase-independent apoptosis through activation of apoptosis-inducing factor in breast cancer cells. Apoptosis.

[12] Freeman SM, Abboud CN, Whartenby KA, Packman CH, Koeplin DS, Moolten FL, Abraham GN (1993). The “bystander effect”: tumor regression when a fraction of the tumor mass is genetically modified. Cancer Res.

[13] Kumar NM, Gilula NB (1996). The gap junction communication channel. Cell.

[14] Haass NK, Wladykowski E, Kief S, Moll I, Brandner JM (2006). Differential induction of connexins 26 and 30 in skin tumors and their adjacent epidermis. J Histochem Cytochem.

[15] Xu T, Zhang S, Zheng L, Yin L, Xu L, Peng J (2012). A 90-day subchronic toxicological assessment of dioscin, a natural steroid saponin, in Sprague-Dawley rats. Food Chem Toxicol.

[16] Shim SH, Lee EJ, Kim JS, Kang SS, Ha H, Lee HY, Kim C, Lee JH, Son KH (2008). Rat growth-hormone release stimulators from fenugreek seeds. Chem Biodivers.

[17] Gu L, Tao X, Xu Y, Han X, Qi Y, Xu L, Yin L, Peng J (2016). Dioscin alleviates BDL- and DMN-induced hepatic fibrosis via Sirt1/Nrf2-mediated inhibition of p38 MAPK pathway. Toxicol Appl Pharmacol.

[18] Holden PR, McGuire B, Stoler A, Balmain A, Pitts JD (1997). Changes in gap junctional intercellular communication in mouse skin carcinogenesis. Carcinogenesis.

[19] Laird DW, Fistouris P, Batist G, Alpert L, Huynh HT, Carystinos GD, Alaoui-Jamali MA (1999). Deficiency of connexin43 gap junctions is an independent marker for breast tumors. Cancer Res.

[20] Haass NK, Smalley KS, Herlyn M (2004). The role of altered cell-cell communication in melanoma progression. J Mol Histol.

[21] Stoletov K, Strnadel J, Zardouzian E, Momiyama M, Park FD, Kelber JA, Pizzo DP, Hoffman R, VandenBerg SR, Klemke RL (2013). Role of connexins in metastatic breast cancer and melanoma brain colonization. J Cell Sci.

[22] Trosko JE, Ruch RJ (1998). Cell-cell communication in carcinogenesis. Front Biosci.

[23] Saito-Katsuragi M, Asada H, Niizeki H, Katoh F, Masuzawa M, Tsutsumi M, Kuniyasu H, Ito A, Nojima H, Miyagawa S (2007). Role for connexin 26 in metastasis of human malignant melanoma: communication between melanoma and endothelial cells via connexin 26. Cancer.

[24] Yang J, Liu TJ, Jiang YX, Lu Y (2012). ATRA enhances the bystander effect of suicide gene therapy driven by the specific promoter LEP 503 in human lens epithelial cells. Mol Vis.

[25] Carystinos GD, Katabi MM, Laird DW, Galipeau J, Chan H, Alaoui-Jamali MA, Batist G (1999). Cyclic-AMP induction of gap junctional intercellular communication increases bystander effect in suicide gene therapy. Clin Cancer Res.

[26] Park JY, Elshami AA, Amin K, Rizk N, Kaiser LR, Albelda SM (1997). Retinoids augment the bystander effect *in vitro* and *in vivo* in herpes simplex virus thymidine kinase/ganciclovir-mediated gene therapy. Gene Ther.

[27] Jin ZJ (2004). About the evaluation of drug combination. Acta Pharmacol Sin.

