# Supplementary material for: Dioscin augments HSV-tk-mediated suicide gene therapy for melanoma by promoting connexin-based intercellular communication
Source: Oncotarget. 2016 Nov 26;8(1):798–807. doi: 10.18632/oncotarget.13655 (PMC5352197; doi:10.18632/oncotarget.13655)
Supplement: Supplementary file 1 [file oncotarget-08-798-s001.pdf]

Dioscin augments HSV-tk-mediated suicide gene therapy for melanoma by promoting connexin-based intercellular communication

Supplementary Materials

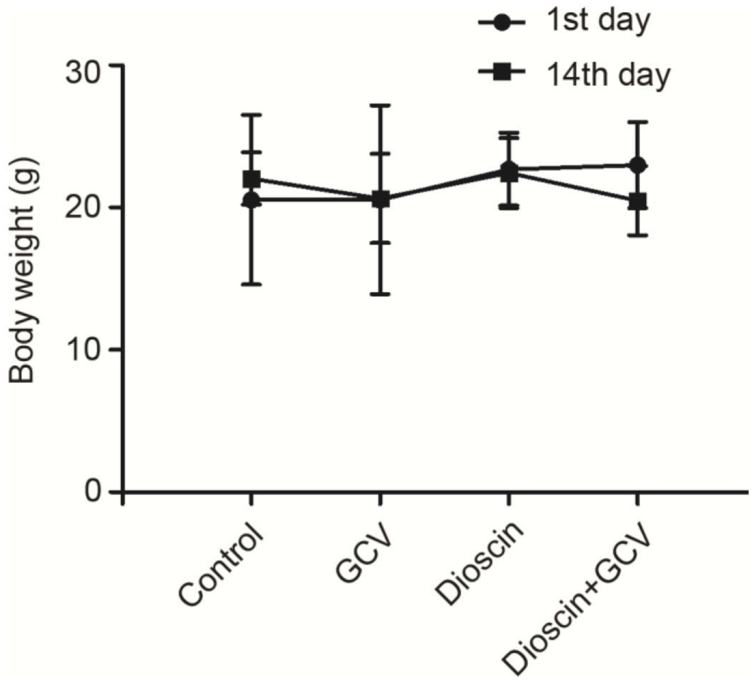

Supplementary Figure S1: Body weight of the mice. Mice bearing B16 tumors were randomized into four groups (*n* = 14 mice per group): control, (saline only), GCV only (100 mg/kg•day), dioscin only (50 mg/kg•day), and GCV plus dioscin. Body weight of the mice was measured on day 1 and on day 14, respectively. Data are presented as the mean ± standard deviation.
